# Mesophyll conductance is unaffected by expression of Arabidopsis *PIP1* aquaporins in the plasmalemma of *Nicotiana*

**DOI:** 10.1093/jxb/erac065

**Published:** 2022-02-20

**Authors:** Victoria C Clarke, Annamaria De Rosa, Baxter Massey, Aleu Mani George, John R Evans, Susanne von Caemmerer, Michael Groszmann

**Affiliations:** Division of Plant Sciences, Research School of Biology, The Australian National University, Acton, Australian Capital Territory 2601, Australia; Division of Plant Sciences, Research School of Biology, The Australian National University, Acton, Australian Capital Territory 2601, Australia; Division of Plant Sciences, Research School of Biology, The Australian National University, Acton, Australian Capital Territory 2601, Australia; Division of Plant Sciences, Research School of Biology, The Australian National University, Acton, Australian Capital Territory 2601, Australia; University of Essex, UK

**Keywords:** Carbon isotope discrimination, mesophyll conductance, *Nicotiana tabacum*, photosynthesis, PIP aquaporin genes, transgenic

## Abstract

In plants with C_3_ photosynthesis, increasing the diffusion conductance for CO_2_ from the substomatal cavity to chloroplast stroma (mesophyll conductance) can improve the efficiencies of both CO_2_ assimilation and photosynthetic water use. In the diffusion pathway from substomatal cavity to chloroplast stroma, the plasmalemma and chloroplast envelope membranes impose a considerable barrier to CO_2_ diffusion, limiting photosynthetic efficiency. In an attempt to improve membrane permeability to CO_2_, and increase photosynthesis in tobacco, we generated transgenic lines in *Nicotiana tabacum* L. cv Petite Havana carrying either the Arabidopsis *PIP1;2* (*AtPIP1;2*) or *PIP1;4* (*AtPIP1;4*) gene driven by the constitutive dual 2x35S CMV promoter. From a collection of independent T_0_ transgenics, two T_2_ lines from each gene were characterized, with western blots confirming increased total aquaporin protein abundance in the *AtPIP1;2* tobacco lines. Transient expression of *AtPIP1;2-mGFP6* and *AtPIP1;4-mGFP6* fusions in *Nicotiana benthamiana* identified that both AtPIP1;2 and AtPIP1;4 localize to the plasmalemma. Despite achieving ectopic production and correct localization, gas exchange measurements combined with carbon isotope discrimination measurements detected no increase in mesophyll conductance or CO_2_ assimilation rate in the tobacco lines expressing *AtPIP*. We discuss the complexities associated with trying to enhance *g*_m_ through modified aquaporin activity.

## Teaser

Arabidopsis aquaporins AtPIP1;2 and AtPIP1;4 whose genes were ectopically expressed in tobacco localized to the plasma membrane but did not detectably increase mesophyll conductance.

## Introduction

Enhancing photosynthetic processes has increasingly been a research target due to the need to improve crop yields to feed a growing global population in the face of changing climates and diminishing resources (Ray *et al.*, 2013; Bailey-Serres *et al.*, 2019). A key first step in C_3_ photosynthesis is the diffusion of atmospheric CO_2_ into leaves where it is fixed by Rubisco within chloroplasts. Improving the conductance to CO_2_ diffusion within leaves is predicted to increase photosynthetic capacity and ultimately crop yields, while also improving water use efficiency (Lundgren and Fleming, 2020). Several points of resistance for CO_2_ diffusion occur on the path from atmosphere into chloroplasts (Clarke *et al.*, 2021). Initially, CO_2_ diffuses through the leaf boundary layer and stomatal pores, whose aperture limits the ease with which CO_2_ passes into the substomatal cavity and regulates water loss from the leaf. CO_2_ diffusing between substomatal cavity airspaces and mesophyll tissue encounters resistance from the cell wall, plasma membrane, cytosol, chloroplast envelope, and stroma (the aqueous chloroplast phase). The sum of all these resistances is termed mesophyll resistance, and its inverse, mesophyll conductance (*g*_m_), captures how efficiently CO_2_ can move through mesophyll tissue to the chloroplast stroma where it is fixed by Rubisco.

We know little about the exact resistance to CO_2_ associated with the cell membranes. Experiments using artificial membranes found that CO_2_ diffuses rapidly through simple lipid bilayers, arguing that there is no need for facilitated transmembrane transport of CO_2_. However, subsequent work highlighted that unlike simple lipid bilayers, biological membranes have high protein and sterol content that substantially reduces their permeability to CO_2_, suggesting the need for embedded membrane channels or transporters (Endeward *et al*., 2014, 2017). However, others argue that the solubility–diffusion model (also known as the Meyer–Overton rule) alone still accounts for gaseous CO_2_ transfer across biological membranes (Missner and Pohl, 2009). Modelling of the diffusion resistances in plants, suggests that plant cell membranes represent a significant component of *g*_m_ in leaves (Evans *et al*., 1994, 2009; Tholen and Zhu, 2011; von Caemmerer and Evans, 2015; Evans, 2021), and that factors that increase membrane CO_2_ permeability should increase *g*_m_ and consequently CO_2_ assimilation rate.

Manipulation of genes to alter the transmembrane protein composition of biological membranes to facilitate CO_2_ diffusion has been an active area of research over the past 25 years. Nakhoul *et al.* (1998) first reported that heterologous expression of *human AQUAPORIN 1* (*hAQP1*) in *Xenopus* oocytes increased plasma membrane permeability to CO_2_. Aquaporins (AQPs), such as hAQP1, are pore-forming membrane-spanning proteins belonging to the larger Major Intrinsic Protein (MIP) family. Originally named after their ability to passively move water across membranes, AQPs have since been reported to facilitate the transfer of many different substrates across biological membranes, including gases (Maurel *et al.*, 2015). Several members of the Plasma membrane Intrinsic Protein (PIP) subfamily, which are homologous to hAQP1, have evidence of facilitating CO_2_ transport across cell membranes (Uehlein *et al.*, 2017). Consistent with a role in regulating *g*_m_, PIP proteins generally localize to the plasma membrane, with some isoforms also detected in chloroplast envelopes by western blot and proteomic analysis (Uehlein *et al.*, 2008; Beebo *et al.*, 2013).

Using *Xenopus* ooctyes and yeast heterologous systems, NtAQP1, also known as NtPIP1;5s (De Rosa *et al.*, 2020; Groszmann *et al.*, 2021), was the first plant AQP identified to be permeable to CO_2_ (Uehlein *et al.*, 2003; Otto *et al.*, 2010). Since then, a number of other CO_2_-permeable PIPs have been identified using heterologous systems, including AtPIP1;2 (Heckwolf *et al.*, 2011), AtPIP2;1 (Wang *et al.*, 2016), *Hordeum vulgare* HvPIP2;1, HvPIP2;2, HvPIP2;3 and HvPIP2;5 (Mori *et al.*, 2014), *Zea mays* ZmPIP1;5 and ZmPIP1;6 (Heinen *et al.*, 2014), and *Setaria italica* SiPIP2;7 (Ermakova *et al.*, 2021).

*In planta* studies revealed that decreasing *NtAQP1/NtPIP1;5s* transcript abundance by RNA interference (RNAi), resulted in tobacco plants with reduced photosynthetic rate and mesophyll conductance (Flexas *et al.*, 2006; Uehlein *et al.*, 2008). Similarly, T-DNA knock-out mutants of an Arabidopsis homolog, *AtPIP1;*2, and co-suppression of rice *Oryza sativa OsPIP* genes also resulted in a reduced *g*_m_ (Hanba *et al.*, 2004; Heckwolf *et al.*, 2011). On the other hand, Kromdijk *et al.* (2019) failed to observe any difference in *g*_m_ between wild-type (WT) and single knockout mutants of *AtPIP1;2*, *AtPIP1;3* or *AtPIP2;6* in Arabidopsis.

Conversely, there are reported examples where overexpression of PIP AQPs has improved photosynthesis. Overexpression of *NtAQP1/NtPIP1;5s* in its native host tobacco led to an increase in *g*_m_ by 20% compared with controls, with a corresponding increase in CO_2_ assimilation rate (Flexas *et al.*, 2006). Similarly, overexpression of *SiPIP2;7* increased *g*_m_ and CO_2_ assimilation in the C_4_ photosynthetic species *S. italica* (Ermakova *et al.*, 2021), while overexpression of *OsPIP1;2* increased *g*_m_ and photosynthesis, and improved productivity in rice (Xu *et al.*, 2019). Cross-species expression of some AQP isoforms have also been shown to increase mesophyll conductance including, tobacco *NtAQP1/NtPIP1;5s* in Arabidopsis (Sade *et al.*, 2014) and tomato (Kelly *et al.*, 2014), barley *HvPIP2;1* in rice (Hanba *et al.*, 2004), and *Mesembryanthemum crystallinum, McMIPB* (PIP1-subtype), in tobacco (Kawase *et al.*, 2013). Thus, the use of foreign AQPs represents another potential avenue to engineer improvements in *g*_m_ and CO_2_ assimilation in species of interest.

Tobacco is a popular model species that is closely related to crops of economic interest such as tomatoes, potatoes, eggplants, and peppers, and itself has renewed commercial applications in the biofuel and plant-based pharmaceutical sectors. Tobacco is capable of scaling from the laboratory to the field and as such is a key model for trialing transgenic manipulations to improve photosynthesis before translation into food crops. In this study, we investigated the effects on membrane permeability to CO_2_ in tobacco plants expressing the Arabidopsis PIP1 AQPs *AtPIP1;2* and *AtPIP1;4*. We confirm protein expression and subcellular localization to the plasma membrane of tobacco mesophyll cells for these AtPIPs, but could not detect an increase in mesophyll conductance. We discuss the complexities associated with trying to enhance *g*_m_ through modified aquaporin activity.

## Materials and methods

### Assembly of constructs

*AtPIP1;2* and *AtPIP1;4*, and *β-glucuronidase* (*GUS*) (non-AQP expression control) protein-coding sequences (CDS) were commercially synthesized (Genscript) as gateway-enabled entry vectors (i.e. included flanking attL sites) incorporating dicot optimal Kozak translation start site sequences (AGAACC**ATG**GAA). A second set of *AtPIP1;2* and *AtPIP1;4* genes without the stop codon were also made, for use in green fluorescent protein (GFP) C-terminal fusion constructs. The *AtPIP1;2*, *AtPIP1;4*, and *GUS* CDSs were cloned into expression vectors of the pMDC Gateway-compatible *Agrobacterium* sp. binary vector system (Curtis and Grossniklaus, 2003) using Gateway *LR Clonase II* enzyme mix (Thermo Fisher Scientific). Full length CDSs (i.e. including stop codon) were inserted into pMDC32 (MG0100 in this study) resulting in a final cassette of RB-2x35S:AtPIP1;2:nosT-Hyg^r^-LB, RB-2x35S:AtPIP1;4:nosT-Hyg^r^-LB, and RB-2x35S:GUS:nosT-Hyg^r^-LB. AtPIP CDSs without the stop codons were cloned into pMDC83 (MG0101 in this study) to create C-terminal GFP fusions with the final cassette being RB-2x35S:AtPIP1;2-GFP6his:nosT-Hyg^r^-LB and RB-2x35S:AtPIP1;4- GFP6his:nosT-Hyg^r^-LB. All *E. coli* cloning steps used One Shot OmniMAX 2 T1R Chemically Competent *E. coli* cells (Thermo Fisher Scientific). All final plasmids were Sanger sequenced to confirm accuracy of the clones using Wizard Plus SV Minipreps DNA Purification Systems (Promega), BigDye sequencing chemistry (Thermo Fisher Scientific), and ZR DNA Sequencing Clean-Up Kit (Zymo Research). Final expression vectors were transformed via electroporation into *Agrobacterium tumefaciens* strain GV3101 and PCR genotyped with primers flanking the *AtPIP* or *GUS* CDS (2x35S forward (fwd): 5ʹ-TCATTTGGAGAGGACCTCGA; NOS-term: 5ʹ-GCAAGACCGGCAACAGGATT).

### Stable transgenic plants

Surface-sterilized seeds of WT *Nicotiana tabacum*, L. cv Petite Havana were germinated in sterile Magenta boxes containing half-strength Murashige and Skoog (MS) medium (pH 5.7), 3% (w/v) sucrose and 0.3% (w/v) agarose and grown for 4 weeks at 28 °C at 350 ± 100 μmol photons m^−2^ s^−1^ illumination and 16 h daylength. Four-week-old tobacco plants were used for transformation via the leaf disc method on co-cultivation medium (Horsch *et al.*, 1985). Leaf discs were then transferred to regeneration medium consisting of MS-based medium containing agar (0.6 g l^−1^), hygromycin (50 mg l^−1^), timentin (50 mg l^−1^), 6-benzylaminopurine (BAP; 1 mg l^−1^), 1-naphthaleneacetic acid (NAA; at 1 mg l^−1^), *myo*-inositol (100 mg l^−1^), and thiamine (1 mg l^−1^), for 2–3 weeks to stimulate callus formation. Thereafter, the calli were transferred to fresh regeneration medium (agar (0.6 g l^−1^), hygromycin (50 mg l^−1^), timentin (50 mg l^−1^), BAP (1 mg l^−1^), NAA (1 mg l^−1^), *myo*-inositol (100 mg l^−1^) and thiamine (1 mg l^−1^)) to stimulate shoot growth. Prominent shoots were excised and placed onto rooting plates consisting of MS agar medium with hygromycin (50 mg l^−1^) and timentin (50 mg l^−1^). Ten hygromycin-resistant primary transformants for each construct (T_0_ generation) with established root systems were transferred to soil and allowed to self-fertilize. The presence of the transgene was confirmed via PCR genotyping on gDNA using the above 2x35S fwd and NOS-term reverse primer set.

### qPCR to quantify transgene expression

T_1_ generation seedlings were selected on hygromycin (50 mg l^−1^) MS-based agar medium for 18 d post-germination at 16 h light, 22 °C and light intensities between 100 and 120 μmol m^−2^ s^−1^. Three biological replicates per independent transgenic line, each consisting of aerial tissue from five T_1_ seedlings, were harvested and snap frozen in liquid nitrogen. Tissue was ground using a Qiagen TissueLyser II and RNA extracted using the ISOLATE II RNA Plant Kit (Meridian Bioscience). RNA was quality checked using a NanoDrop spectrophotometer (Thermo Fisher Scientific) and diluted to 200 ng μl^−1^. One microgram of RNA was aliquoted and DNaseI treated as per the manufacturer’s instructions (cat. no. 18068015; Thermo Fisher Scientific). cDNA was generated using the sensiFAST cDNA Synthesis Kit (Meridian Bioscience) as per the manufacturer’s instructions and diluted 1:10. Real-time reactions were set up using SensiFAST SYBR Lo-ROX Kit (Meridian Bioscience) chemistry as per the manufacturer’s instructions using 1 μl of cDNA (~5 ng). qPCR reactions were performed in a 384-well plate format on a ViiA 7 Real-Time system (Applied Biosystems/Thermo Fisher Scientific) using the cycle format: 95 °C, 2 min (×1); 95 °C, 5 s; 60 °C, 10 s; 72 °C, 10 s (×40); and finished with a melt curve between 95 °C and 60 °C. QuantStudio Real-Time PCR Software (Thermo Fisher Scientific) was used to capture and analyse the data. Primers specific to the 3ʹ end of the AtPIP CDS and within the 3ʹ transcribed region of the NOS-terminator were used to detect transgene abundance; AtPIP1;2 (fwd: 5ʹ-TTGCTGCTCTCTACCACGT; and reverse (rev): 5ʹ-GAAATTCGAGCTCCACCGC) and AtPIP1;4 (fwd: 5ʹ-TCTAGCAGCACTATATCACCAGA; and rev: 5ʹ-GAAATTCGAGCTCCACCGC). Data were analysed using the Δ*C*_T_ method, with *NtUBC2* expression used for normalization (Schmidt and Delaney, 2010) (fwd: 5ʹ-AGCTGCTATACTGACTTCAATCCA; and rev: 5ʹ-TCTCACTGAACATGCGTGCT).

### Transient expression in *Nicotiana benthamiana*

Wild-type *Nicotiana benthamiana* plant were grown for 4 weeks in a CONVIRON (Winnipeg, Canada) growth chamber under a 16 h/8 h day/night cycle at 28 °C/22 °C with 60% humidity and with approximately 100 μmol photons m^−2^ s^−1^ light intensity. Agroinfiltraion was performed as described in Rolland (2018). Briefly, *Agrobacterium tumefacians* GV3101(pMP90) (Koncz and Schell, 1986) was transformed with plasmids containing C-terminal GFP fusions with *AtPIP1;2* or *AtPIP1;4* and grown on LB medium containing rifampicin (50 μg ml^−1^) and kanamycin (25 μg ml^−1^). Cultures were grown at 28 °C with shaking at 220 rpm. *Nicotiana benthamiana* leaves were co-infiltrated on their abaxial surface with either AtPIP1;2-GFP or AtPIP1;4-GFP vectors and a vector containing the P19 protein to inhibit post-translational gene silencing and allow the PIP–GFP fusion constructs to be expressed (Roth *et al.*, 2004).

### Protoplast preparation and confocal microscopy

Leaf sections of approximately 4 cm×4 cm were harvested 3 d post-infiltration from *N. benthamiana* leaves transformed with either AtPIP1;2-GFP + P19 or AtPIP1;4-GFP + P19. Protoplasts were isolated as detailed in Rolland *et al.* (2016). In two independent experiments, around 100 protoplasts (per independent experiment) expressing GFP-tagged constructs were observed, and a selection were imaged using an upright Zeiss LSM780 confocal laser-scanning microscope (Carl Zeiss), a ×40 water immersion objective (NA 1.1) and the Zen Blue software package (Carl Zeiss). GFP and chlorophyll were excited at 488 nm and emission recorded at 499–535 nm and 630–735 nm, respectively. Higher resolution images (with the same excitation and emission spectra) were obtained on an upright Zeiss LSM800 with Airyscan (Carl Zeiss) fitted with a ×63 oil immersion objective (NA 1.4), and Zen Blue software package (Carl Zeiss).

Further imaging on selected protoplasts was conducted using the Stellaris 8 Falcon (Leica) utilizing FAST FLIM to separate chlorophyll autofluorescence and GFP signals, under the following conditions: ×40 water lens (NA 1.1), excitation 473 nm, emission 493–550 nm and 10 line accumulations. Additional confocal imaging on the Stellaris excited GFP at 482 nm (emissions at 525–542 nm), and chlorophyll at 650 nm (emission 667–755 nm), using a ×40 water lens (NA 1.1).

### Plant growth

Tobacco (*Nicotiana tabacum*, L. cv Petite Havana) was grown in a naturally lit glasshouse with day/night temperatures set at 28/18 °C in 5-litre pots filled with Debco Green Wizard commercial potting mix supplemented with slow release fertilizer at 7 g l^−1^ (Osmocote Exact, Scotts, NSW, Australia). Plants were grown between October and November 2019 in Canberra (Australia), and watered daily. Average light intensity at midday during the growing period was 1400 µmol m^−2^ s^−1^.

### Gas exchange measurements

CO_2_ response curves of CO_2_ assimilation rate and chlorophyll fluorescence were measured together with a LI-6800 portable photosynthesis system (LI-COR Biosciences, USA) at a leaf temperature of 25 °C, irradiance of 1500 µmol quanta m^−2^ s^−1^, relative humidity of 55%, 21% O_2_ and varying reference CO_2_ concentrations (0, 50, 75, 100, 200, 300, 400, 600, 800, 1000, 1200 µmol mol^−1^). All gas exchange measurements were made on the youngest expanded leaf of 4-week-old plants. Curves were analysed to derive estimates of maximum Rubisco activity, *V*_cmax,_ and the rate of electron transport, *J* (Sharkey *et al.*, 2007). *V*_cmax_ was estimated from measurements below *C*_i_=400 µbar and *J* was estimated from measurements between *C*_i_=400 and 800 µbar. Triose phosphate utilization (TPU) was not assigned as a limitation in the calculations of *J*. Direct measurements of *g*_m_ (0.5 mol m^−2^ s^−1^ bar^−1^) from carbon isotope discrimination measurements on the same plants (see below) were used when fitting the model.

### Concurrent measurements of gas exchange and carbon isotope discrimination to quantify mesophyll conductance

Gas exchange and carbon isotope discrimination measurements were made as described by Tazoe *et al.* (2011) using a 6 cm^2^ chamber of the LI-6400 with a red blue light emitting diode (LED) light source (LI-COR). Two LI-6400 chambers and the plants were placed in a temperature-controlled cabinet with fluorescent lights (TRIL1175, Thermoline Scientific Equipment, Smithfield, NSW, Australia). The CO_2_ in the leaf chamber was set at 380 µmol mol^−1^, flow rate at 200 µmol s^−1^ and irradiance at 1500 µmol quanta m^−2^ s^−1^. Leaf temperature was controlled at 25 °C. N_2_ and O_2_ were mixed by mass flow controllers (Omega Engineering Inc., Stamford, CT, USA) to generate 2% O_2_, which was supplied to the LI-6400s after humidification of the air by adjusting the temperature of water circulating around a Nafion tube (Perma Pure LLC, Toms River, NJ, USA, MH-110-12P-4). Gas exchange was coupled to a tunable diode laser (TDL; TGA100a, Campbell Scientific, Inc., Logan, UT, USA) for concurrent measurements of carbon isotope composition. Measurements were made at 4-min intervals for 20 s, with 10–12 measurements per leaf and the last five measurements were averaged. The δ^13^C of CO_2_ gas cylinders (δ^13^C_tank_) used in the LI-6400 CO_2_ injector system was −10.5 ± 0.5‰. Gas exchange was calculated using the equations presented by von Caemmerer and Farquhar (1981) and Δ was calculated from the equation presented by Evans *et al.* (1986). The average value of ξ was 6.9 with a standard deviation of 2.52, where ξ=*C*_ref_/(*C*_ref_−*C*_sam_) and *C*_ref_ and *C*_sam_ are the CO_2_ concentrations of dry air entering and exiting the leaf chamber, respectively, measured by the TDL. Measurements were taken on four 6-week-old plants on the youngest expanded leaf. Mesophyll conductance, *g*_m_, was calculated as described by Evans and von Caemmerer (2013).

### Western blots

To isolate protein from leaves, leaf discs of 0.71 cm^2^ corresponding to area where gas exchange was measured were collected and frozen immediately in liquid N_2_. One disc was ground in ice-cold glass homogenizer in 0.5 ml of protein extraction buffer: 100 mM Tris–HCl, pH 7.8, supplemented with 25 mM NaCl, 20 mM EDTA, 2% SDS (w/v), 10 mM dithiothreitol, and 2% (v/v) protease inhibitor cocktail (Sigma, St Louis, MO, USA). Protein extracts were incubated at 65 °C for 10 min and then centrifuged at 13 000 *g* for 1 min at 4 °C to obtain clear supernatant. Protein extracts were diluted into 4× SDS Sample buffer containing 0.25M Tris–HCl pH 6.8, 40% (v/v) glycerol, 8% SDS, 4% bromophenol blue, 0.5% β-mercaptoenthanol, and incubated at 95 °C for 5 min. Samples were loaded on a leaf area basis and separated by polyacrylamide gel electrophoresis (Nu-PAGE 4–12% Bis-Tris gel, Thermo Fisher Scientific) in running buffer (pH 7.3) containing 50 mM MES, 50 mM Tris, 0.1% SDS (w/v), 20 mM EDTA. Proteins were transferred to a nitrocellulose membrane and probed with antibodies against PIP proteins (Agrisera cat. no. AS09487, Vännäs, Sweden) at 1:1000 dilution. Quantification of western blots was performed with Image Lab software (Bio-Rad Laboratories, Hercules, CA, USA).

### Statistical analysis

All statistical analyses were performed using two-way analysis of variance. Comparison of means was made using a 0.05 significance level using Tukey’s *post hoc* test (OriginPro 2020, OriginLab Corp.).

## Results

Ten independent T_0_ transgenic tobacco (cv Petite Havana) lines were generated for both the *2x35S:AtPIP1;*2 and *2x35S:AtPIP1;4* transgenes. PCR genotyping using primers specific to the AtPIP transgenes confirmed their presence in the T_0_ lines. Positive T_0_ tobacco lines were allowed to self-pollinate and produce T_1_ seeds. The T_1_ lines were sown on hygromycin medium to select for progeny carrying the transgene. Segregation counts were used to indicate transgene locus number, and RNA extracted from five pooled 18-day-old seedlings was used to profile *AtPIP* transgene expression (see Supplementary Fig. S1). We selected *AtPIP1;*2 T_1_ lines 6 (single insertion) and 10 (double insertion), and *AtPIP1;4* T_1_ lines 5 (double insertion) and 10 (single insertion) for preliminary physiological analysis. *AtPIP1;2* T_1_ line 6 and *AtPIP1;4* T_1_ line 5 showed increased photosynthetic and mesophyll conductance and T_2_ lines derived from *AtPIP1;2* line 6 (single insertion) and *AtPIP1;4* line 5 (double insertion) were then analysed further.

**Fig. 1.**
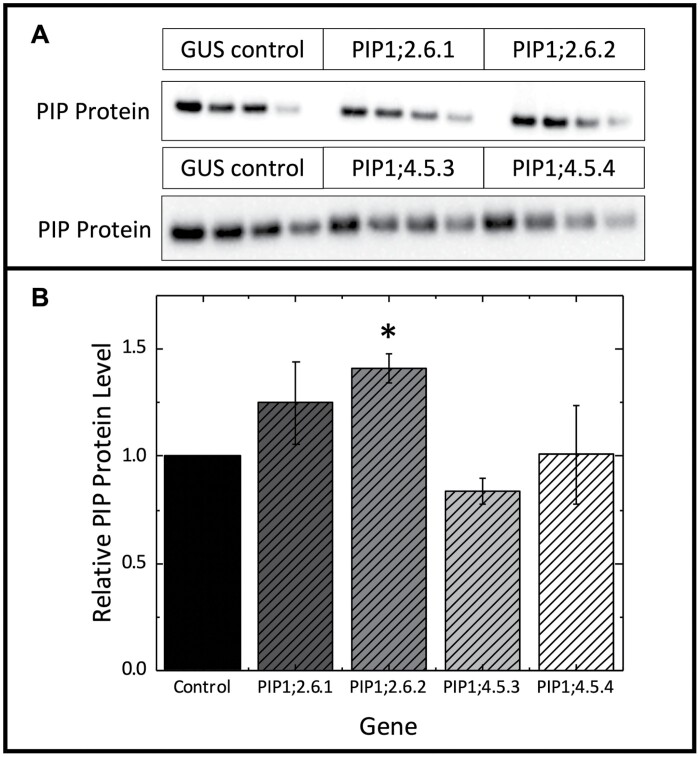
Analysis of AtPIP overexpression lines. (A) Western blot dilution series at 80, 65, 50, and 35% loading for GUS transgenic control and T_2_ ectopic expression lines of AtPIP1;2.6.1, AtPIP1;2.6.2, AtPIP1;4.5.3, and AtPIP1;4.5.4 with an anti-PIP1 antibody (Agisera) which reacts to both native tobacco PIP1s and Arabidopsis PIP1s. (B) Only the AtPIP1;2.6.2 expressing line had significantly more overall PIP protein than the control (*n*=3, two way analysis of variance with *post hoc* Tukey test, *P*<0.05).

AtPIP protein levels in T_2_ lines were assayed in leaf tissue collected from 6-week-old plants by western blot with a α-PIP antibody, which is reported (Agrisera, Sweden) to react to all five AtPIP1 proteins (AtPIP1;1, AtPIP1;2, AtPIP1;3, AtPIP1;4, and AtPIP1;5) and potentially, but less likely, AtPIP2 proteins as the epitope is located within the C loop that is somewhat conserved between PIP1 and PIP2. In non-AtPIP expressing control plants (a stable *2x35S:*GUS line, used as a surrogate WT transgenic control), a western signal was detected at 28 kDa, consistent with the expected size of the PIP1 monomer. This cross-reactivity with NtPIPs is unsurprising given the close structural conservation of PIP1 proteins across dicot angiosperms (De Rosa *et al.*, 2020). A faint band was also detected at approximately 50 kDa, which likely corresponds to undenatured PIP dimers (not shown). Lines expressing *AtPIP1;2* had significantly increased PIP protein signal over control plants, while lines expressing *AtPIP1;4* were not significantly different from controls (Fig. 1).

**Fig. 2.**
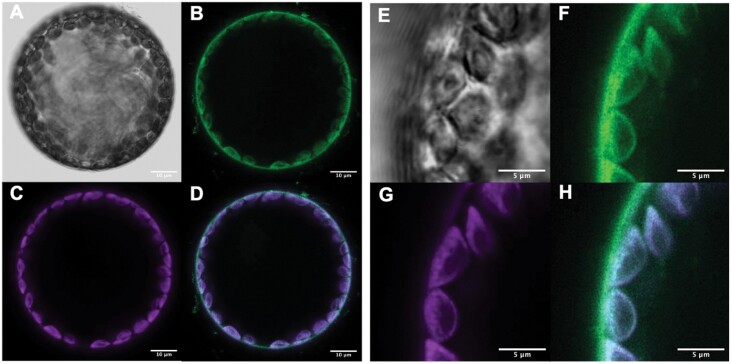
Subcellular localization of the AtPIP1;2–mGFP fusion protein in *Nicotiana benthamiana* mesopyhyll cells, under excitation at 488 nm. AtPIP1;2-mGFP localizes to the plasma membrane. Whole protoplast (A–D) (imaged on a Ziess 780 confocal microscope; scale bars, 10 µm) and highlighted details at higher magnification (E–G) (imaged on a Zeiss 800 Airyscan; scale bars, 5 µm). (A, E) Brightfield; (B, F) GFP signal (green, emission: 499–535 nm); (C, G) chlorophyll autofluorescence (magenta, emission 630–735 nm); (D, H) composite images of GFP and chlorophyll autofluorescence channels. GFP signal is distinctly present on the plasma membrane, but bleed-through of chlorophyll autofluorescence into the GFP-channel is present on the chloroplast envelope and within the chloroplast (white).

**Fig. 3.**
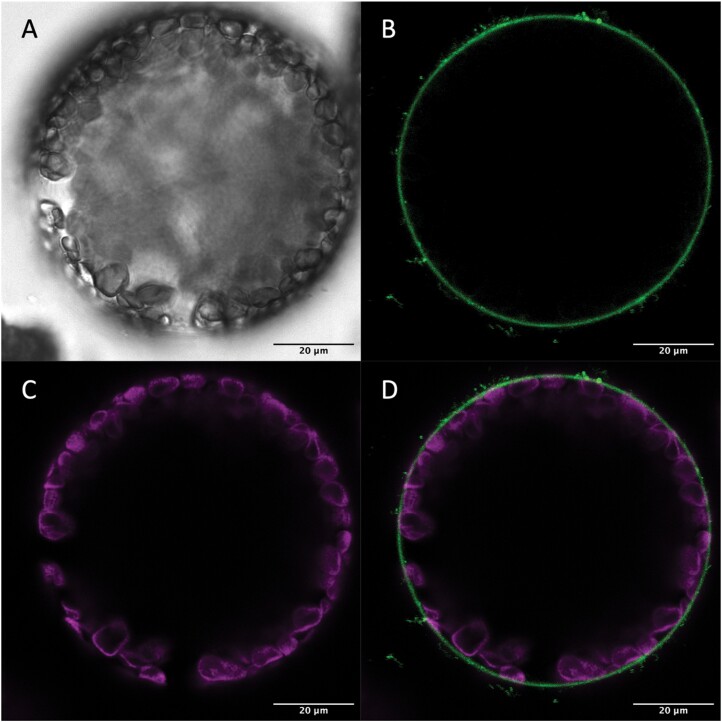
Subcellular localization of the AtPIP1;4-mGFP fusion protein in *Nicotiana benthamiana* mesophyll cells, with GFP excitation at 482 nm. AtPIP1;4-mGFP localized to the plasma membrane. (A–D) Whole protoplast imaged on a Leica Stellaris Falcon confocal microscope (scale bars, 20 µm). (A) Brightfield; (B) GFP signal (green; excitation at 482 nm, emission 525–542 nm); (C) chlorophyll autofluorescence (magenta; excitation at 650 nm, emission 667–755 nm); (D) a composite image of GFP and chlorophyll autofluorescence channels. Under these excitation and emission conditions, no chlorophyll bleed-through at the chloroplast was observed in the GFP channel.

Subcellular localization patterns of the AtPIP1 proteins in tobacco were determined using C-terminal GFP fusions driven by the 2x35S promoter and transiently expressed in the close relative of tobacco, *Nicotiana benthamiana* (Schiavinato *et al.*, 2020). When imaged under confocal microscopy at 488 nm excitation, the GFP-tagged AtPIP proteins were observed in the green channel (499–535 nm), while the autofluorescence from chlorophyll was captured in the magenta channel (630–735 nm). AtPIP1;2-GFP and AtPIP1;4-GFP each localized to the extreme periphery of the cell, consistent with plasma membrane localization (Figs 2, 3). Signal was also detected in the GFP channel associated with the chloroplast and chloroplast envelope of isolated tobacco mesophyll protoplasts (Fig. 2). To determine if the chloroplast envelope signal was PIP–GFP or chlorophyll bleed-through into the GFP channel, isolated protoplasts were imaged with Fluorescence Lifetime Imaging (FAST FLIM, Leica Stellaris Falcon). GFP signal was observed only on the plasma membrane for both AtPIP1;2–GFP and AtPIP1:4–GFP (see Supplementary Fig. S2). Further, the optimal excitation and emission wavelengths for PIP–GFP were determined using a lambda lambda scan (Stellaris Falcon, Leica), and imaging of AtPIP1;4–GFP under these conditions eliminated chlorophyll bleed-through into the GFP channel (Fig. 3).

**Fig. 4.**
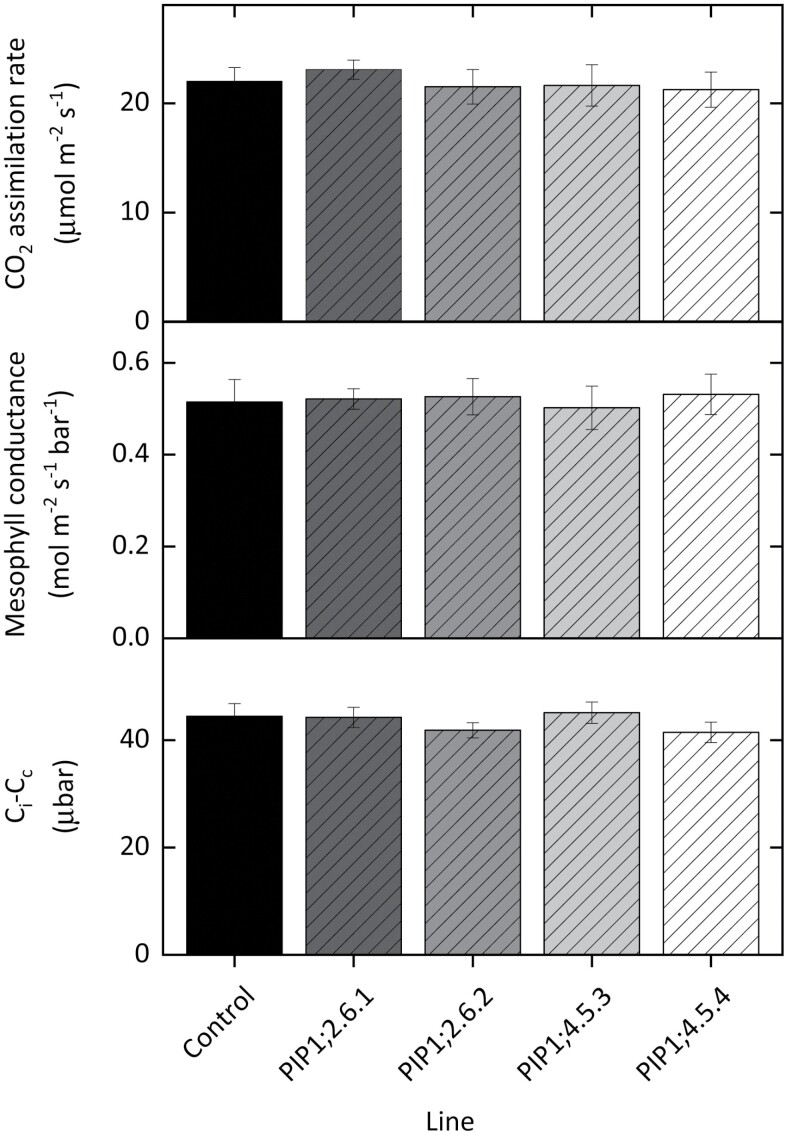
Expression of *AtPIP1;2* or *AtPIP1;4* did not affect CO_2_ assimilation rate (A), mesophyll conductance (B), or the draw-down of CO_2_ into the chloroplasts (*C*_i_−*C*_c_) (C) compared with control plants (black). Measurements taken at 25 °C, 380 ppm CO_2_ and 2% O_2_, *n*=4. No significant differences were observed between any lines (two-way analysis of variance, *P*>0.05).

**Fig. 5.**
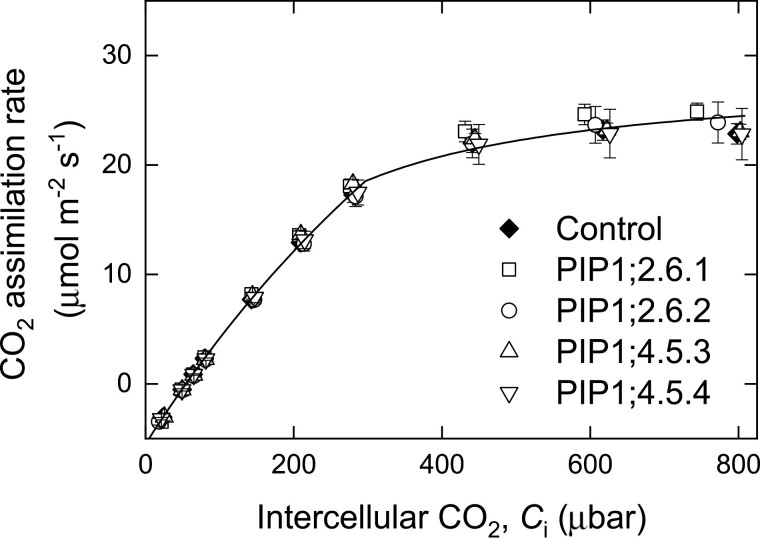
CO_2_ response curves of lines expressing *AtPIP1* (open symbols) were comparable (no significant differences) to the control (solid symbol). Measurements taken at 25 °C and 21% O_2_, *n*=4. No significant differences were observed between any lines (two-way analysis of variance, *P*>0.05).

Two T_2_ progeny of single insertion line #6 of *AtPIP1;2* (lines 6.1 and 6.2) and two from double insertion line #5 of *AtPIP1;4* (lines 5.3 and 5.4) were grown for physiological analysis. The *AtPIP1;2* and *AtPIP1;4* lines showed similar leaf mass per area to the GUS control lines (Table 1). Steady state CO_2_ assimilation rates (measured at 300 ppm CO_2_) were comparable to GUS controls (Fig. 4A). Mesophyll conductance and the draw-down of CO_2_ into the chloroplasts (*C*_i_−*C*_c_) was also unchanged in the AtPIP1;2 and AtPIP1;4 transgenics (Fig. 4B, C). The CO_2_ response of assimilation was measured, and the expression of the *AtPIP1;2* or *AtPIP1;4* transgene did not significantly increase assimilation rates (Fig. 5A). *V*_cmax_ values derived from gas exchange data and our sequential measurements of mesophyll conductance were not significantly different from controls (Table 1). Electron transport rate (*J*) calculated from gas exchange data was not significantly different from controls for any of the *AtPIP1* transgenic lines (Table 1).

**Table 1.** Summary of physiological and biochemical parameters measured and modelled for *AtPIP1* overexpression lines in tobacco

**Parameter**	**GUS control**	**PIP1;2.6.1**	**PIP1;2.6.2**	**PIP1;4.5.3**	**PIP1;4.5.4**
Hygromycin copy number	0	2	2	2–4	2–4
PIP protein levels (relative signal)	1.00	1.25 ± 0.19	1.41 ± 0.07*	0.78 ± 0.06	1.02 ± 0.23
Leaf mass per area (g m^−2^)	25.9 ± 2.0	25.8 ± 1.5	23.3 ± 1.5	21.0 ± 1.9	22.7 ± 1.4
Steady state assimilation rate (μmol m^−2^ s^−1^)	21.6 ± 1.2	23.1 ± 0.9	21.5 ± 1.6	21.6 ± 1.9	21.3 ± 1.6
Steady state *C*_i_ (380 ppm CO_2_) (μbar)	211.0 ± 3.2	204.6 ± 3.0	208.8 ± 6.5	200.5 ± 5.7	204.9 ± 7.2
Stomatal conductance (CO_2_) (mol m^−2^ s^−1^)	0.30 ± 0.03	0.29 ± 0.01	0.29 ± 0.02	0.27 ± 0.05	0.27 ± 0.01
Mesophyll conductance (mol m^−2^ s^−1^ bar^−1^)	0.50 ± 0.04	0.52 ± 0.02	0.53 ± 0.04	0.50 ± 0.05	0.53 ± 0.04
*V*_cmax_ (gas exchange) (μmol m^−2^ s^−1^)	85.1 ± 5.2	90.5 ± 3.6	81.5 ± 8.0	90.0 ± 3.5	84.3 ± 9.1
*J* (gas exchange) (μmol m^−2^ s^−1^)	111.5 ± 4.9	124.5 ± 3.4	118.3 ± 11.7	114.8 ± 3.3	113.5 ± 13.7

Steady state assimilation, steady state *C*_i_, stomatal conductance, and mesophyll conductance measurements were made at 380 ppm CO_2_ and 2% O_2_. *V*_cmax_ and *J* (gas exchange) were calculated with the model of Sharkey *et al.* (2007). *Significant difference from control (*n*=3, *P*<0.5)

## Discussion

Crop plants like tobacco are characterized by relatively thin cell walls, and analysis suggests that membranes may represent up to 50% of the overall mesophyll CO_2_ diffusion resistance in these leaves (Evans *et al.*, 1994; von Caemmerer and Evans, 2015; Clarke *et al.*, 2021; Evans, 2021). Increasing membrane permeability to CO_2_ is one way to increase *g*_m_ and subsequently photosynthetic rates. Improving *g*_m_ would also improve drought tolerance in plants and be complementary to other improvements of photosynthesis, such as increasing the efficiency of Rubisco and RuBP regeneration (Long *et al.*, 2015). A subset of PIP AQP isoforms have been identified as capable of permeating CO_2_, which has led to research investigating the role AQPs play in facilitating CO_2_ diffusion across the mesophyll plasma membrane and chloroplast envelope and their potential for engineering improvements in photosynthesis (for review see Groszmann *et al.*, 2017).

There are several examples where overexpression of PIP aquaporins has resulted in increases in *g*_m_ (see Introduction). Overexpression of *NtAQP1* in tobacco led to an increase in *g*_m_ by 20% compared with controls, with a corresponding increase in CO_2_ assimilation rate (Flexas *et al.*, 2006). However, the increase in CO_2_ assimilation rate unexpectedly occurred at higher *C*_i_ and no changes in initial slope of the CO_2_ response curves were observed (Flexas *et al.*, 2006). Expression of the PIP proteins HvPIP2;1 or OsPIP1;2 in rice also increased *g*_m_ (Hanba *et al.*, 2004; Xu *et al.*, 2019). AtPIP1;2, which is a close homolog of the CO_2_ transporting NtAQP1/NtPIP1;5s, shows evidence of influencing *g*_m_ in its native Arabidopsis. This includes; *Atpip1;2* T-DNA knock-out mutants having reduced *g*_m_ (Heckwolf *et al.*, 2011) and a 50% reduction in the CO_2_ permeability of chloroplasts (Tolleter *et al.*, 2017), and overexpression of *AtPIP1;2* in the *Atpip1;2* mutant background restoring *g*_m_ back to WT levels (Heckwolf *et al.*, 2011). More recently, however, Kromdijk *et al.* (2019) failed to replicate the mutant observations, with no differences observed in *g*_m_ between WT and the single lines for *AtPIP1;2* (or *AtPIP1;3* and *AtPIP2;6*) in Arabidopsis. Our results are somewhat analogous in that we did not observe any differences in mesophyll conductance when ectopically expressing Arabidopsis *AtPIP1;2* in tobacco, but knock-down and ectopic expression studies are not directly comparable and are subject to other influencing factors.

We worked on transgenic lines with the greatest transgene expression. Ectopic expression of *AtPIP1* in tobacco was driven by the 2x35S promoter, which has previously successfully driven ectopic expression of tobacco AQP1 in tomato to phenotype (Kelly *et al.*, 2014). qRT-PCR data showed that our transgenes were expressed (Supplementary Fig. S1), and our confocal data confirmed protein production and incorporation of AtPIP1;2–GFP and AtPIP1;4–GFP into the plasma membrane (Figs 2, 3). The localization of AQPs to the chloroplast envelope through confocal microscopy is complicated by the difficulty in distinguishing GFP fluorescence from chlorophyll autofluorescence at the chloroplast, as observed here in Fig. 2. This can be resolved through spectral unmixing or fluorescence lifetime imaging techniques. Here, lifetime imaging clearly showed no AtPIP1;2–GFP or AtPIP1;4–GFP signal was present at the chloroplast envelope (see Supplementary Fig. S2). With optimized excitation and emission wavelengths, bleed-through of chlorophyll autofluorescence into the GFP channel can also be eliminated (Fig. 3). A plasma membrane localization of AtPIP1;2 and AtPIP1;4 is consistent with the localization pattern of the *g*_m_-enhancing OsPIP1;2, with OsPIP1;2-GFP localizing to the plasma membrane in rice protoplasts derived from culms (stems) of dark grown plants (Xu *et al.*, 2019). In Xu *et al* (2019) confocal images of rice culm cells expressing OsPIP1;2, fluorescence is evident around an internal structure that was not specified, but is likely an etioplast (differentiating chloroplast), but without further analysis it is not clear if this is a true GFP signal. PIP proteins have previously been detected in the chloroplast envelope by proteomics (Kleffmann *et al.*, 2004; Ferro *et al.*, 2010; Simm *et al.*, 2013), but contamination from plasma and vacuolar membranes cannot be excluded (Beebo *et al.*, 2013). Uehlein *et al.* (2008), using immuno-gold labelling in tobacco, reported plasma membrane localization for NtAQP1/NtPIP1;5s and gold particles were also observed on the chloroplast envelope indicating NtAQP1 at least is present on the chloroplast envelope in tobacco.

Quantifying AQP protein content was complicated by the close homology of PIPs across species and the inevitable cross-reactivity of α-PIP antibodies to both the transgenic Arabidopsis and native tobacco PIPs. We chose not to attach an epitope tag to our *AtPIP1;2* and *AtPIP1;4* transgenes for fear they may obscure the channel passage, given that both the N- and C-terminal tails reside adjacent to the cytosolic channel opening in the tertiary structure, and are important steric regulators of PIP channel activity (reviewed in Groszmann *et al.*, 2017; Tyerman *et al.*, 2021). Western blots indicated a modest increase of up to 40% in leaf PIP AQP protein content above controls in the *AtPIP1;2* overexpressing lines. Total PIP protein content was similar between control and *AtPIP1;4* lines, despite active transgene expression. As we cannot distinguish between tobacco and Arabidopsis PIPs, it is possible that the native NtPIP isoforms could have been down-regulated in response to ectopic AtPIP1;4 production. Interaction between ectopically expressed PIP proteins with native tobacco PIP proteins is an important consideration as PIP2 proteins are involved in the recruitment of PIP1 proteins to the plasma membrane (see Groszmann *et al.*, 2017). As AtPIP1;2 was detected on the plasma membrane, we can assume it must be interacting with the native PIP2 proteins and forming heterodimers. It is possible, however, that these cross-species heterodimers have altered functionality and may not transfer CO_2_ across the membrane.

We used combined measurements of gas exchange and carbon isotope discrimination, which is one of the most robust techniques for quantifying *g*_m_ (Pons *et al.*, 2009). The plants were well-watered with values of *g*_m_ for our control tobacco lines (0.5 mol m^−2^ s^−1^ bar^−1^) similar to our previous measurements of WT tobacco based on carbon isotope discrimination (Evans *et al.*, 1994; Yamori *et al.*, 2010; von Caemmerer and Evans, 2015; Clarke *et al.*, 2021). Despite all of the above, we did not detect an improved *g*_m_ phenotype. There are several factors that may have influenced a change in phenotype and we discuss these below.

**Fig. 6.**
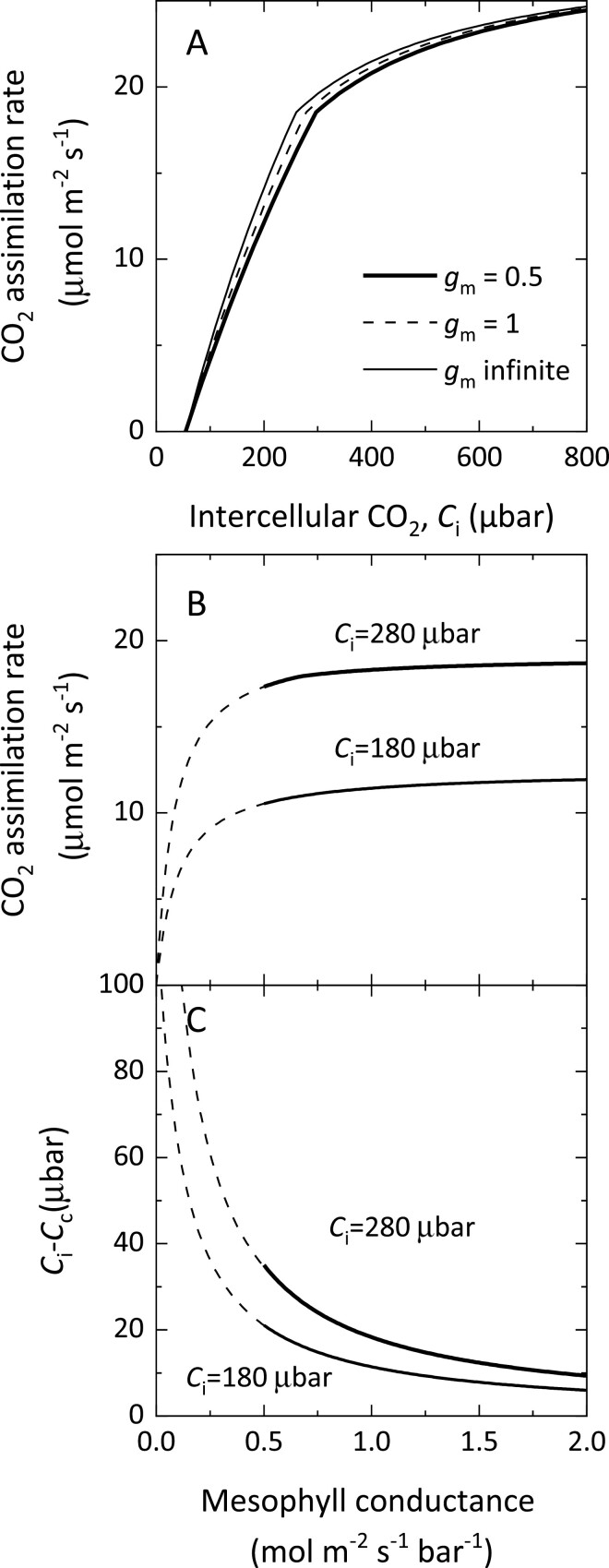
Modelled responses to mesophyll conductance of CO_2_ assimilation rate and the drawdown in CO_2_ partial pressure from the intercellular airspace to the chloroplast. (A) CO_2_ response curves observed with *g*_m_ of 0.5 mol m^−2^ s^−1^ bar^−1^, double and infinite *g*_m_. (B) CO_2_ assimilation rates as a function of *g*_m_ at intercellular CO_2_, *C*_i_, of 280 and 180 µbar. (C) Drawdown in CO_2_ partial pressure between the intercellular airspaces (*C*_i_) and the sites of carboxylation in the chloroplast (*C*_c_). The model curves were generated with the Farquhar, von Caemmerer, and Berry model (von Caemmerer, 2000) at 25 °C and 21% O_2_ with the Rubisco kinetic constants given in Sharkey *et al.* (2007). Model curves assume Rubisco activity of 80 μmol m^−2^ s^−1^, electron transport rate *J*=123 μmol m^−2^ s^−1^, and a respiration rate *R*_d_=2.3 μmol m^−2^ s^−1^. In (B, C), modelled curves at 280 µbar are electron transport limited, whereas the curves at 180 µbar are Rubisco limited.

Modelling of various gas-exchange parameters (Fig. 6), suggested that at our high basal *g*_m_ values for controls (0.5 mol m^−2^ s^−1^ bar^−1^), it would have been more difficult to statistically detect differences in the CO_2_ response curves (Fig. 6A) or increased CO_2_ assimilation rates (Fig. 6B) because of the diminishing increases in these traits per unit improvement in *g*_m_. However, differences in *g*_m_ and *C*_i_*−C*_c_ would have likely been apparent (Fig. 6C). Our *g*_m_ values are greater than *g*_m_ values reported by Flexas *et al.* (2006) (0.32 mol m^−2^ s^−1^ bar^−1^) and the exceptionally low values reported by Kawase *et al.*, (2013) (0.108 mol m^−2^ s^−1^ bar^−1^). However, these differences are not surprising as it is well known that photosynthetic rate and *g*_m_ vary with growth conditions (Evans and von Caemmerer, 1996; Yamori *et al.*, 2010). Our higher basal *g*_m_ values, which are expected in agricultural systems, would therefore make detection of improvements more difficult than those observed in systems with lower basal *g*_m_ values (Fig. 6).

Tobacco is a recently emerged allotetraploid and its highly duplicated genome encodes 84 AQP genes, of which 30 belong to the PIP subfamily (Groszmann *et al.*, 2021). For comparison, the Arabidopsis genome encodes for only 35 AQPs in total, with 13 being PIPs (Quigley *et al.*, 2001). *NtAQP1/NtPIP1;5s* and its sister gene *NtPIP1;5t* are expressed equally and are by far the most abundantly expressed AQPs in tobacco leaves (De Rosa *et al.*, 2020). This highlights the possibility that higher transgene expression is required in tobacco to sufficiently exceed native PIP mRNA levels in order to affect *g*_m_ compared with other species, such as Arabidopsis.

Our understanding of the effects of individual resistance components on CO_2_ transfer capacity is limited by our inability to accurately isolate and measure the impact of each component. However, recent studies have helped to refine our estimations of these resistance components, through anatomical measurements and mutant analyses (Tosens *et al.*, 2012; Clarke *et al.*, 2021; Evans, 2021). The plasma membrane and chloroplast envelope appear to account for around half of the mesophyll CO_2_ diffusion resistance in crop species with thin mesophyll cell walls, such as tobacco (Evans *et al.*, 1994; Yamori *et al.*, 2010; von Caemmerer and Evans, 2015; Clarke *et al.*, 2021; Evans, 2021). However, our modelling shows that significant increases in membrane-associated CO_2_ conductance improve overall mesophyll conductance by a much smaller fraction (see Supplementary Fig. S3). For example, a 50% increase in CO_2_ conductance across the membranes would yield only a 20% improvement in mesophyll conductance (Supplementary Fig. S3). This modelling is supported by experimental data, such as Flexas *et al.* (2006), where doubling PIP protein levels increased mesophyll conductance by only 40%.

We observed an increase of up to 40% in PIP proteins in the membranes of the mesophyll cells (AtPIP1;2.6.2, Fig. 1), and assuming this was all active and functional, our modelling suggests this would improve *g*_m_ by ~16% which translates into an increase from our basal *g*_m_ of 0.50 to 0.58 mol m^2^ s^−1^ bar^−1^ (see Supplementary Fig. S3). This is only slightly greater than the error rate (biological and technical replication errors) of *g*_m_ in this study of around 10% (Supplementary Fig. S3; Table 1). We might therefore be achieving an increase in *g*_m_, but it is indistinguishable from the background variation of our measurements.

The normal basal *g*_m_ value may be an important factor influencing the ease with which *g*_m_ might be improved through transgenic engineering. Environmental factors during the growth of plants (e.g. photoperiod, light intensity, day/night temperature, nutrient supply, watering, and humidity) can impact anatomical and biochemical traits that determine *g*_m_. Lipid and protein composition of membranes can be strongly remodelled in response to environmental cues (Uemura *et al.*, 1995). Under growth conditions that lead to a higher basal *g*_m_, increasing *g*_m_ further through engineering becomes more challenging. For instance, Kelly *et al.* (2014) failed to observe an improvement in *g*_m_ when overexpressing *NtAQP1* in tomato until basal *g*_m_ values were tempered by overexpressing hexokinase (*AtHXK1*). Conditional effects also seem to plague the understanding of PIPs in their native roles in *g*_m_ and CO_2_ assimilation through mutant analysis. Although several studies show that a loss of CO_2_-permeable PIPs reduces *g*_m_ (Hanba *et al.*, 2004; Flexas *et al.*, 2006; Uehlein *et al.*, 2008; Heckwolf *et al.*, 2011), other studies have failed to corroborate these findings, with the contrasting results interpreted as differences in growth conditions between studies (Kromdijk *et al.*, 2020). Recently such conditional responses (growth conditions and growth stage) between *PIP* loss-of-function mutants and declines in photosynthetic rates and *g*_m_ have been observed in rice (Huang *et al.*, 2021), and in tomato a *SlPIP1;2* knockout mutant only reduced *g*_m_ when mutants were grown under CO_2_ enrichment (Zhang *et al.*, 2021).

It appears that the conditional relevance of PIPs and their involvement in regulating *g*_m_ and photosynthetic rates requires further study in order to more intricately assess and improve the consistency of engineering efforts. To detect an increase in *g*_m_, we may need to investigate different growth conditions. Higher transgene expression appears a strong necessity but may be an overly simplistic view. We have a limited understanding of the capacity of cellular membranes to support additional integral membrane proteins, along with the composition and regulation of the AQP tetramers. The assembly of AQPs into functional tetramers is complex and the specific monomers and their ratios can influence substrate specificity (Jozefkowicz *et al.*, 2017). For instance, increasing ratios of NtAQP1 over NtPIP2;1 in a tetrameric complex gradually switches specificity from water to CO_2_ with mingled transport specificities in between (Otto *et al.*, 2010). PIP AQPs can also move in and out of membranes in response to environmental cues, and their channel activity and substrate specificity is regulated by phosphorylation gating mechanisms (Groszmann *et al.*, 2017; Qiu *et al.*, 2020). Phosphomimetic versions may help ensure transgenic PIPs remain in their desired transport state and embedded within membranes (Qiu *et al.*, 2020).

## Conclusion

Ectopic expression of Arabidopsis aquaporins *AtPIP1;2* and *AtPIP1;4* in the plasma membrane of tobacco mesophyll cells did not increase mesophyll conductance to CO_2_, or other photosynthetic parameters. While it has been shown that some PIP AQP isoforms are capable of transporting CO_2_ in heterologous systems, translation of this capability to plants to improve *g*_m_ and CO_2_ assimilation has had varied results. Plant growth and environmental conditions may play a significant role in the ability for AQPs to alter *g*_m_. Further studies are needed to better understand aquaporin function. These could include conditional transgene expression, co-expression of PIPs to induce desirable heterodimers/tetramers or alterations to phosphorylation states to enhance channel activation and membrane integration.

## Supplementary data

The following supplementary data are available at *JXB* online.

Fig. S1. Transgene expression in T_1_ lines.

Fig. S2. Lifetime imaging of GFP and chlorophyll fluorescence signals in mesophyll cells expressing AtPIP1;4-GFP localization construct.

Fig. S3. Modelled effect of increased membrane conductance of CO_2_ on total mesophyll conductance.

erac065_suppl_Supplementary_Figures_S1-S3

## Data Availability

All data supporting the findings of this study are available within the paper and within its supplementary materials published online.
