# Supplementary material for: Mesophyll conductance is unaffected by expression of Arabidopsis PIP1 aquaporins in the plasmalemma of Nicotiana
Source: J Exp Bot. 2022 Feb 20;73(11):3625–36. doi: 10.1093/jxb/erac065 (PMC9162178; doi:10.1093/jxb/erac065)
Supplement: erac065_suppl_Supplementary_Figures_S1-S3 [file erac065_suppl_supplementary_figures_s1-s3.pdf]

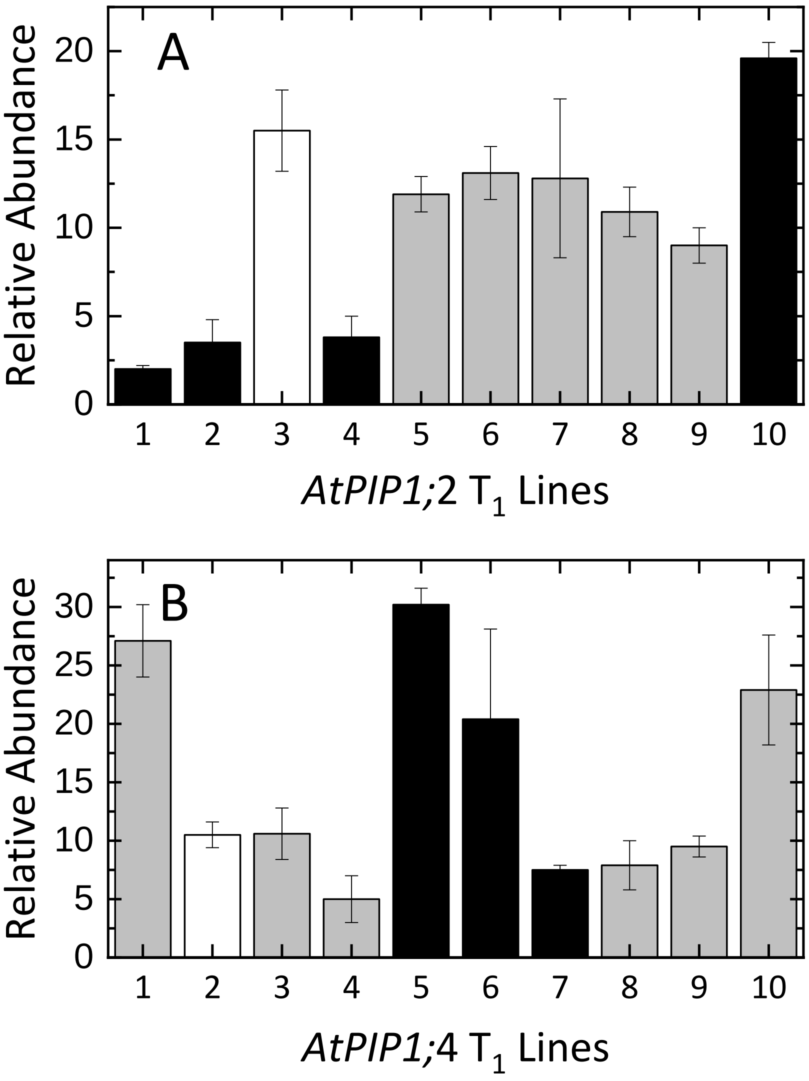

Supp Fig. S1.

Relative qRT-PCR gene expression in 10 independent T_1 lines expressing either *AtPIP1;2* (**A**) or *AtPIP1;4* (**B**), generated with transgene specific primers. Relative abundance is the fold change normalised to the *NtUBC2* housekeeping gene. n = 3 biological replications, each consisting of aerial tissue of 5 pooled 18-day old T_1 hygromycin selected seedlings. Insertion number was determined by segregation counts (n = 30-100 seedlings). Black bars indicate two insertions, grey bars indicate one insertion and white bars indicate an indeterminate insertion number.

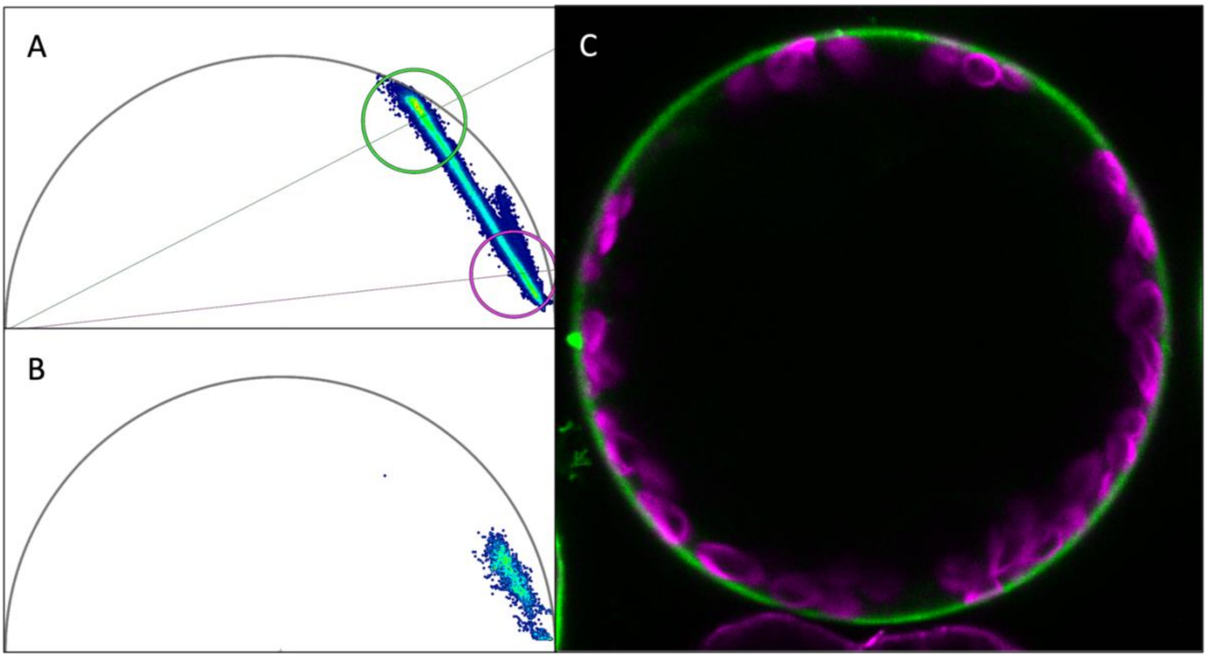

Supp Fig. S2.

A Fluorescence lifetime imaging (FLIM) phasor plot of the AtPIP1;4-mGFP fusion protein in a *Nicotiana benthamiana* mesophyll cell, showing two distinct signal populations corresponding to chlorophyll (magenta) and GFP (green). **B** Fluorescence lifetime imaging phasor plot of an untransformed *Nicotiana benthamiana* mesophyll cell, showing lifetime signal for chlorophyll fluorescence only. Phasor plots are graphical representations of all the raw fluorescence lifetime imaging (FLIM) data in a vector space, with each pixel in the FLIM image transformed to a point in the phasor plot. **C** Component separated image based on lifetime signals in AtPIP1;4-mGFP fusion protein in a *Nicotiana benthamiana* mesophyll cell (corresponding to **A**). AtPIP1;4-GFP signal is present only on the plasma membrane. Comparable data was also obtained for AtPIP1;2-GFP fusion proteins (not shown).

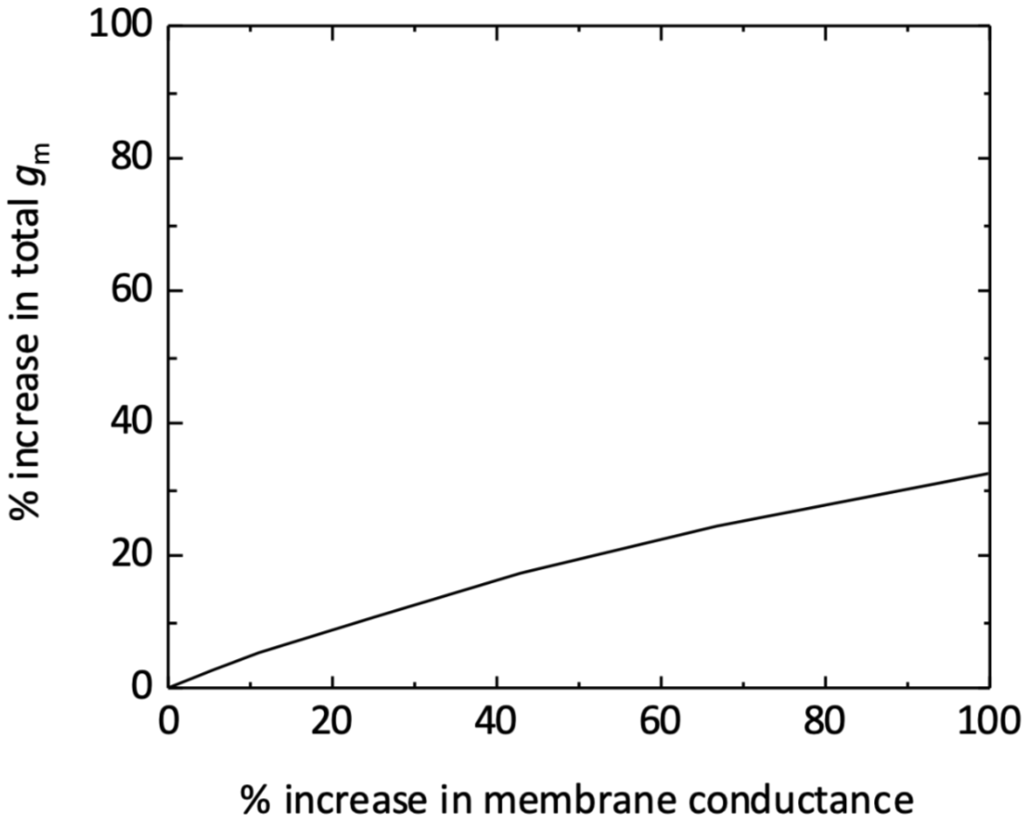

Supp Fig. S3.

Modelled effect of increased membrane conductance to CO_2 and the effect on total mesophyll conductance. Calculated from Tobacco data where resistance imposed by the plasmalemma and chloroplast membranes accounts for 49% of total CO_2 resistance in mesophyll cells (Evans *et al.*, 1994). The model is based on data from Evans *et al* (1994). The equation is $\frac{1}{g_m} = \frac{1}{g_{mem}} + \frac{1}{g_{wall}} + \frac{1}{g_{liquid}}$, where g_{mem}, g_{wall} and g_{liquid} denoted the conductance across the membrane, the cell wall and across the liquid phase.
